# Development and Validation of Amisulpride in Human Plasma by HPLC Coupled with Tandem Mass Spectrometry and its Application to a Pharmacokinetic Study

**DOI:** 10.3797/scipharm.1105-12

**Published:** 2011-07-25

**Authors:** Ramakotaiah Mogili, Kanchanamala Kanala, Balasekhara Reddy Challa, Babu Rao Chandu, Chandrasekhar Kottapalli Bannoth

**Affiliations:** 1 Jawaharlal Nehru Technological University, Anantapur, Andhra Pradesh, 515002, India; 2 Siddhartha Institute of Pharmaceutical Sciences, Jonnalagadda, Narasaraopet, Guntur, Andhra Pradesh, 522601, India; 3 Nirmala College of Pharmacy, Madras Road, Kadapa, Andhra Pradesh, 516002, India; 4 Donbosco PG College of Pharmacy, Guntur, Andhra Pradesh, India

**Keywords:** Liquid chromatography–tandam mass spectrometry, Method development, Method Validation, Amisulpride

## Abstract

In this study, authors developed a simple, sensitive and specific liquid chromatography–tandem mass spectrometry (LC–MS/MS) method for quantification of Amisulpride in human plasma using Amisulpride-*d*_5_ as an internal standard (IS). Chromatographic separation was performed on Zorbax Bonus-RP C18, 4.6 × 75 mm, 3.5 μm column with an isocratic mobile phase composed of 0.2% formic acid:methanol (35:65 v/v), at a flow-rate of 0.5 mL/min. Amisulpride, Amisulpride-*d*_5_ was detected at m/z 370.1→242.1 and 375.1→242.1. The drug and the IS were extracted by a liquid-liquid extraction method. The method was validated over a linear concentration range of 2.0–2500.0 ng/mL for Amisulpride with a correlation coefficient of (r^2^) ≥ 0.9982. This method demonstrated intra- and inter-day precision within 0.9 to 1.7 and 1.5 to 2.8 % and intra- and inter-day accuracy within 98.3 to 101.5 and 96.0 to 101.0 % for Amisulpride. Amisulpride was found to be stable at 3 freeze–thaw cycles, bench top and auto sampler stability studies. The developed method was successfully applied to a pharmacokinetic study.

## Introduction

**Fig. 1:**
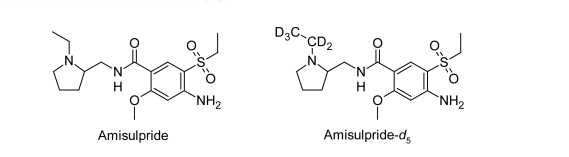
Chemical structures of Amisulpride and Amisulpride-*d*_5_

Amisulpride, 4-amino-N-[(1-ethylpyrrolidin-2-yl)methyl]-5-(ethylsulfonyl)-2-methoxybenzamide, has a molecular formula of C_17_H_27_N_3_O_4_S and a molecular weight of 369.48 g/mol (Fig. 1) [1]. Amisulpride is an *ortho*-methoxybenzamide derivative chemically related to sulpiride. This CNS agent shows a high affinity for dopamine D2 and D3 receptors and demonstrates anti schizophrenic and antidysthymic (antidepressant) properties [2, 3]. This drug is effective as maintenance therapy in patients with chronic schizophrenia, and generally a long-term treatment with Amisulpride was associated with improvement in quality of life and social functioning [4]. Amisulpride is generally well tolerated and the neurological tolerability profile is superior to that of conventional antipsychotics (e.g. haloperidol and flupenthixol) [5, 6]. Amisulpride poisoning may induce seizures, QT prolongation and torsades de pointes [7].

After a single oral dose of 50 mg racemic Amisulpride, the drug shows a rapid absorption with an absolute bioavailability of about 50% and a biphasic absorption profile [8–10]. After a 50 mg oral dose, the renal clearance ranges from about 17–20 l/h and exhibits little variation with the dose administered but correlates linearly with creatinine clearance (the plasma systemic clearance is about 31–421/h) [8–11].

Literature survey reveals that for quantification of amisulpride in biological samples by LC-MS/MS [12–18], HPLC [19–36], pharmaceuticals by chromatographic, electrophoretic, spectrophotometric [37] and voltametric [38] were reported. Of all of the studies, Nirogi et al. [12] achieved better results for quantitative estimation of amisulpride in human plasma. Authors could not achieve better results for quantification of amisulpride in terms of sensitivity, runtime, consumption of mobile phase and in recovery.

The aim of the present research is to develop and validate a simple, sensitive, selective, fast, rugged and reproducible analytical method for quantification of Amisulpride in Human plasma samples by liquid chromatography coupled to electro spray (ES) tandem mass spectrometry. Moreover, the analyte is compared with a deuterated internal standard, which is most useful in selectivity and matrix effect experiments using LC-MS/MS.

## Material and methods

### Standards and chemicals

Amisulpride and Amisulpride-*d*_5_ were obtained from Toronto Research Chemicals Canada. Human plasma (K_2_EDTA) was obtained from Navjeevan Blood Bank, Hyderabad. Formic acid, diethyl ether, methanol and acetone were purchased from SD-Fine Chemicals, Mumbai. Ultra-pure water was obtained from a Milli-Q System.

### Instrumentation

HPLC system (1200 series model, Agilent Technologies, Waldbronn, Germany), Mass spectrometry API 4000 triple quadrupole instrument (ABI-SCIEX, Toronto, Canada) using MRM.

### Detection

Turbo ionspray (API) positive mode with Unit Resolution, MRM was used for the detection of Amisulpride and Amisulpride-*d*_5_. The [MH]^+^ was monitored at m/z: 370.1, for Amisulpride and m/z: 375.2 for Amisulpride-*d*_5_. Fragments of m/z: 242.0 for Amisulpride and m/z: 242.1 Amisulpride-*d*_5_ formed from the respective precursor ions. Mass parameters were optimized as source temperature 500 °C, nebulizer source gas 30 (nitrogen) psi, heater gas 45 (nitrogen) psi, curtain gas 20 (nitrogen) psi, CAD gas 7 (nitrogen) psi, Ion Spray (IS) voltage 5500 volts, source flow rate 500 μL/min without split, entrance potential 10 V, collision cell exit potential (CXP) 12 V, declustering potential (DP) 70 V, Collision energy 38 V for Amisulpride and Amisulpride-*d*_5_.

### Chromatographic conditions

Zorbax Bonus-RP C18, 4.6 × 75 mm, 3.5 μm was selected as the analytical column at a temperature of 30°C. The mobile phase composition was 0.2% formic acid: methanol (35:65 v/v) at a flow rate of 0.5 mL/min. Amisulpride-*d*_5_ was found to be an appropriate internal standard in terms of chromatography and extractability. The retention time of Amisulpride, Amisulpride-*d*_5_ was found to be approximately 1.1 ± 0.2 min.

### Preparation of standards and quality control (QC) Samples

Standard stock solutions of Amisulpride (250.00 μg/mL) and Amisulpride-*d*_5_ (250.00μg/mL) were prepared in methanol. The spiking solution for Amisulpride-*d*_5_ was prepared in 50% methanol from the respective standard stock solution. Standard stock solutions and IS spiking solutions were stored in refrigerator conditions (2–8°C) until analysis. Standard stock solutions were added to drug-free human plasma to obtain Amisulpride concentration levels of 2.0, 4.0, 10.0, 25.0, 250.0, 500.0, 1000.0, 1500.0, 2000.0 and 2500.0 ng/mL for analytical standards and 2.0, 6.0, 750.0 and 1750.0 ng/mL for quality control standards and stored in a −30°C set point freezer until analysis. The aqueous standards were prepared in reconstitution solution (0.2% formic acid: methanol (35:65 v/v) for validation exercises until analysis.

### Sample preparation

Liquid-liquid extraction was used to isolate Amisulpride and Amisulpride-*d*_5_ from human plasma. 100 μL of IS (200.0 ng/mL) and 100 μL of plasma sample (respective concentration) were added respectively into ria-vials and vortexed briefly. Then, 2.5 mL of extraction solvent (diethyl ether) was added and vortexed for approximately 20 min. This was followed by centrifugation at 4000 rpm for 10 min at 20°C. Then samples were flash frozen using dry-ice/acetone. The supernatant from each ria-vial was transferred into another set of ria-vials. These samples were evaporated at 40°C under nitrogen up to dryness. Finally, the dried residue samples were reconstituted with 500 μL of reconstitution solution and vortexed briefly. These samples were transferred into auto sampler vials and injected into LC-MS/MS.

## Method Development and Validation

LC-MS/MS has been used as one of the most powerful analytical tools in clinical pharmacokinetics for its selectivity, sensitivity and reproducibility. The goal of this work is to develop and validate a simple, sensitive, rapid method for quantitative estimation of Amisulpride from plasma samples.

### Mass Spectrometry Optimization

The MS optimization was performed by direct infusion of both the solutions, namely Amisulpride and Amisulpride-*d*_5_, into the mass spectrometer. The critical parameters in the ESI source include the needle voltage and temperature.

Other parameters, such as the nebulizer and heater gases, DP, EP, CE, and CXP were optimized to obtain a better spray shape, resulting in better ionization of the protonated ionic Amisulpride and Amisulpride-*d*_5_. A CAD product ion spectrum of Amisulpride and Amisulpride-*d*_5_ formed with the fragment ions at *m*/*z* 242.1 for Amisulpride and *m*/*z* 242.1 for Amisulpride-*d*_5_ (Fig. 2a–d).

### Chromatographic optimization

Initially, we tried different extraction techniques such as SPE, Precipitation techniques. Finally, LLE was selected as a suitable extraction technique for the drug and IS in terms of recovery and reproducibility. Chromatographic conditions, especially the composition and nature of the mobile phase as well as the column, were optimized through several trials to achieve the best resolution to increase the signal of Amisulpride and Amisulpride-*d*_5_. A good separation and elution were achieved with 0.2% Formic acid: Methanol (35:65v/v) as the mobile phase, at a flow-rate of 0.5 mL/min and injection volume of 5 μL.

The developed method is to be proved by proper validation parameters (selectivity & specificity, linearity, precision & accuracy, recovery, LOQ&LOD, matrix effect and stability) as per standard regulatory guidelines.

#### Selectivity & Specificity

The selectivity of the method was determined by blank human plasma samples from six different lots to test the potential interference of endogenous compounds co-eluted with *the drug* and *IS*. The chromatographic peaks of Amisulpride and Amisulprid-*d*_5_ were identified based on their retention times and MRM responses. The mean peak area of LOQ for Amisulpride and Amisulprid-*d*_5_ at corresponding retention times in blank samples should not be more than 20 and 5% respectively.

#### Linearity, precision and accuracy

The analytical curves were constructed using values ranging from 2.0 to 2500.0 ng/mL for Amisulpride in human plasma. Calibration curves were obtained by weighted 1/conc^2^ quadratic regression analysis. The ratio of Amisulpride peak area to Amisulpride-*d*_5_ peak area was plotted against the ratio of Amisulpride concentration to Amisulpride-*d*_5_ concentration in ng/mL. Calibration curve standard samples were prepared in each set and quality control samples were prepared in replicates (n=6) for analysis. The correlation coefficient >0.9850 was obtained for Amisulpride. Precision and accuracy for the back calculated concentrations of the calibration points, should be within ≤ 15 ± 15% of their nominal values. However, for LLOQ the precision and accuracy should be within ≤ 20 ± 20%.

#### Recovery

The extraction recovery of Amisulpride and Amisulpride-*d*_5_ from human plasma was determined by analyzing quality control samples. Recovery at three concentrations (6.0, 750.0 and 1750.0 ng/mL) was determined by comparing peak areas obtained from the plasma sample and the standard solution spiked with the blank plasma residue. A recovery of more than 50 % was considered adequate to obtain required sensitivity.

#### Limits of Quantification (LOQ) & Detection (LOD)

The response (peak area) was determined in blank plasma samples (six replicates from different plasma) and spiked LOQ sample prepared from the same plasma was determined. The peak area of blank samples should not be more than 20% of the mean peak area of LOQ of Amisulpride, and not more than 5% of Amisulpride. The precision and mean accuracy of the back calculated LOQ replicate concentrations must be ≤ 20 and ± 20%, respectively.

#### Matrix effect

The matrix effect due to the plasma matrix was used to evaluate the ion suppression/enhancement in a signal when comparing the absolute response of QC samples after pretreatment (Liquid-liquid extraction) with the reconstitution samples extracted from blank plasma sample spiking with analyte. Experiments were performed at MQC levels in triplicate with six different plasma lots with the acceptable precision (%CV) of ≤ 15%.

#### Stability (freeze–thaw, auto sampler, bench top, long term)

Low quality control and high quality control samples (n=6) were retrieved from the deep freezer after three freeze-thaw cycles according to the clinical protocol. Samples were frozen at −30°C in three cycles of 24, 36 and 48 h. In addition, the long-term stability of Amisulpride in quality control samples was also evaluated by analysis after 80 days of storage at −30°C. Autosampler stability was studied following a 99 h storage period in the autosampler tray with control concentrations. Bench top stability was studied for a 25 h period with control concentrations. Stability samples were processed and extracted along with the freshly spiked calibration curve standards. The precision and accuracy for the stability samples must be within ≤ 15 and ± 15 % respectively of their nominal concentrations.

#### Analysis of human samples

The bioanalytical method described above was used to determine Amisulpride concentrations in plasma following oral administration to healthy human volunteers. Each volunteer gave written informed consent before participating in this study. Ten healthy volunteers were chosen as subjects and orally administered a 50 mg dose (one 50 mg tablet) with 240 mL of drinking water. The reference product, Solian tablets (Sanofi-Aventis) 50 mg, and the test product, AM tablets (test tablet) 50 mg were used. Study protocol was approved by IEC (Institutional Ethical committee) as per ICMR (Indian Council of Medical Research). Blood samples were collected as pre-dose (0) h, 5 min prior to dosing followed by further samples at 0.25, 0.5, 0.75, 1.0, 1.333, 1.667, 2.0, 2.5, 3.0, 3.5, 4.0, 4.5, 5.0, 5.5, 6.0, 7.0, 9.0, 12.0, 16.0, 24.0, 36.0, 48.0 and 60 h. After dosing, 5 mL of blood was collected each time in vaccutainers containing K_2_EDTA. A total of 48 (24 time points for test and 24 time points for reference) time points were collected from each volunteer. The samples were centrifuged at 3200 rpm, 10°C, 10 min, and stored at −30°C until sample analysis. Test and reference tablets were administered to the same human volunteers under fasting conditions separately with proper washing periods (42 days gap between test and reference doses) as per protocol approved by IEC.

#### Pharmacokinetics and statistical analysis

Pharmacokinetic parameters from the human plasma samples were calculated by a noncompartmental statistics model using WinNon-Lin5.0 software (Pharsight, USA). Blood samples were taken for a period of 3 to 5 times the terminal elimination half-life (t_1/2_) and it was considered as the area under the concentration time curve (AUC) ratio higher than 80% as per FDA guidelines [39–41]. Plasma AM concentration-time profiles were visually inspected and C_max_ and T_max_ values were determined. The AUC_0–t_ was obtained by trapezoidal method. AUC_0-∞_ was calculated up to the last measureable concentration and extrapolations were obtained using the last measureable concentration and the terminal elimination rate constant (K_e_). The terminal elimination rate constant (K_e_) was estimated from the slope of the terminal exponential phase of the plasma of AM concentration–time curve by means of linear regression. The terminal elimination half-life, t_1/2_, was then calculated as 0.693/K_e_. Regarding AUC_0–t_ and C_max_ bioequivalence was assessed by means of analysis of variance (ANOVA) and calculating the standard 90% confidence intervals (90% CIs) of the ratios test/reference (logarithmically-transformed data). The bioequivalence was considered when the ratio of averages of log-transformed data was within 80–125% for AUC_0–t_ and C_max_.

## Results and Discussion

### Selectivity & Specificity

The analysis of Amisulpride and Amisulpride-*d*_5_ using MRM function was highly selective with no interfering compounds. Fig. 3a is showing plasma only for one blank. Chromatograms obtained from plasma (LOQ) spiked with Amisulpride (2.0ng/mL) and Amisulpride-*d*_5_ (200 ng/mL) are shown in Fig. 3b.

### Linearity, Precision and Accuracy

**Fig. 4.**
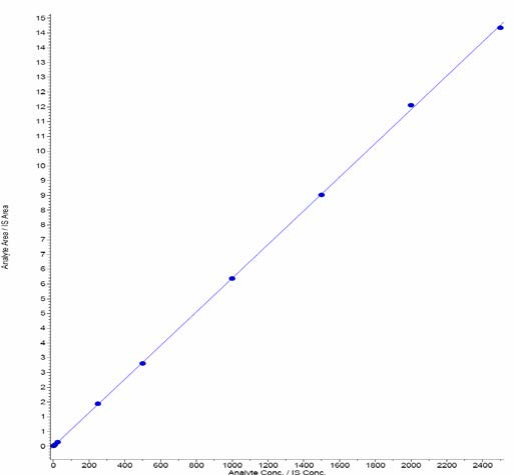
Calibration curve

Calibration curves were plotted as the peak area ratio (Amisulpride / Amisulpride-*d*_5_) versus concentration of Amisulpride. Calibration was found to be linear over the concentration range of 2.0–2500.0 ng/mL for Amisulpride (Fig. 4). The CV% for Amisulpride was less than 3.9%. The accuracy ranged from 96.5 to 101.5% for Amisulpride. The determination coefficient (r^2^) for Amisulpride was greater than 0.9998 for all curves (Table 1). Precision and accuracy for this method were controlled by calculating the intra- and inter-batch variations of QC samples in six replicates at three concentrations (6.0, 750.0 and 1750.0 ng/mL) for Amisulpride as shown in Table 2. The intra-batch CV% was less than 1.7 % for Amisulpride. These results indicate the adequate reliability and reproducibility of this method within the analytical range. This method demonstrated intra- and inter-day Accuracy within 96.0% to 101.5% for Amisulpride.

### Recovery

The recovery following the sample preparation using liquid-liquid extraction method with diethyl ether was calculated by comparing the peak areas of drug in plasma samples with the peak area ratios of solvent samples and was estimated at control levels of drug. The recovery of Amisulpride (at concentrations 6.0, 750.0 and 1750.0 ng/mL) was found to be 74.00, 73.97 and 75.93%. The overall average recovery of Amisulpride was found to be 74.63%. The overall average recovery of Amisulpride-*d*_5_ was found to be 65.07 %.

### Limits of Quantification (LOQ) and Detection (LOD)

The LOQ found for this method is 2.0 ng/ mL with signal-to-noise (S/N) of 30.6. Limit of detection (LOD) was estimated by measuring S/N values obtained in human plasma spiked at 50.0 pg/ mL level and extrapolating the corresponding values to S/N=12.18. The LOD value was 0.25 pg for Amisulpride.

### Matrix effect

The CV % of ion suppression/enhancement in the signal was found to be 1.2% at MQC level for Amisulpride, indicating that the matrix effect on the ionization of the analyte is within the acceptable range under these conditions.

### Stability (Freeze - thaw, Auto sampler, Bench top, Long term)

Quantification of the Amisulpride in plasma subjected to 3 freeze-thaw (−30°C to room temperature) cycles showed the stability of the analyte. The concentrations ranged from 99.0 to 104.0% for Amisulpride of the theoretical values. No significant degradation of the Amisulpride was observed even after a 99 h storage period in the auto sampler tray and the final concentrations of Amisulpride was between 104.83 to 103.94% of the theoretical values. The room temperature stability of Amisulpride in QC samples after 25 h was also evaluated. The concentrations ranged from 94.67 to 102.9% for Amisulpride of the theoretical values. In addition, the long-term stability of Amisulpride in QC samples after 55 days of storage at −30°C was also evaluated. The concentrations ranged from 93.16 to 103.3% for amisulpride of the theoretical values. These results confirmed the stability of Amisulpride in human plasma for at least 55 days at −30°C (Table 3).

### Application to pharmacokinetic study

**Fig. 5.**
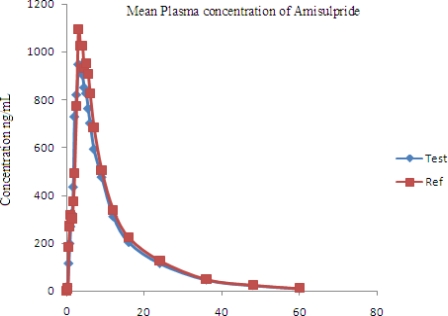
Mean plasma concentrations of test vs. reference after a 50 mg dose (one 50 mg tablet) in 10 healthy volunteers

The above-validated method was used in the determination of AM in plasma samples for establishing the bioequivalence of a single 50-mg dose (one 50 mg tablet) in 10 healthy human volunteers. Typical plasma concentration versus time profiles are shown in Fig. 5. All the plasma concentrations of AM were in the standard curve region and remained above the 2.00 ng mL^−1^ LOQ for the entire sampling period. The pharmacokinetic parameters and 90%CI are shown in Table 4 and 5. Therefore, it can be concluded that the two Amisulpride formulations (reference and test) analyzed were bioequivalent according to regulatory requirements [39–41].

## Conclusion

The method described here is fast, rugged and reproducible, and sensitive. Each sample requires less than 2.5 min of analysis time for determination of Amisulpride in human plasma by LC-MS/MS. The deuterated compound Amisulpride-*d*_5_ was used as an internal standard. We have developed and validated the method as per the FDA guidelines over a concentration range of 2.0–2500.0 ng/mL for Amisulpride using a 100μL plasma sample followed by a simple liquid-liquid extraction technique for extraction of the drug and internal standard. This method could be useful for application in clinical pharmacokinetic studies.

## Figures and Tables

**Fig. 2a.**
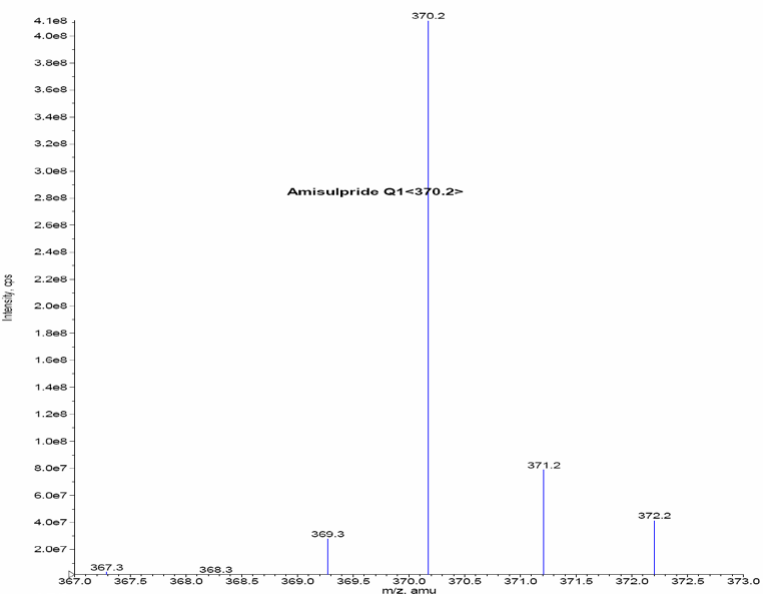
Mass spectrum of Amisulpride parention

**Fig. 2b.**
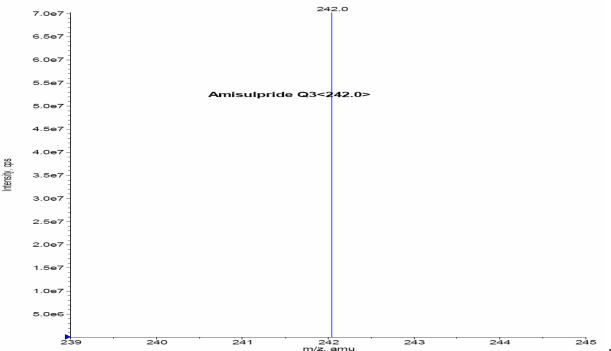
Mass spectrum of Amsulpride production

**Fig. 2c.**
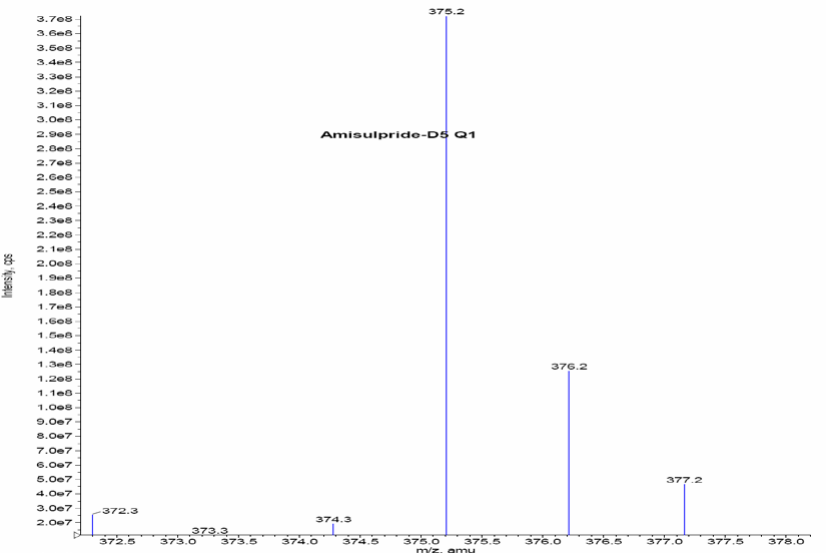
Mass spectrum of Amsulpride-*d*_5_ parention

**Fig. 2d.**
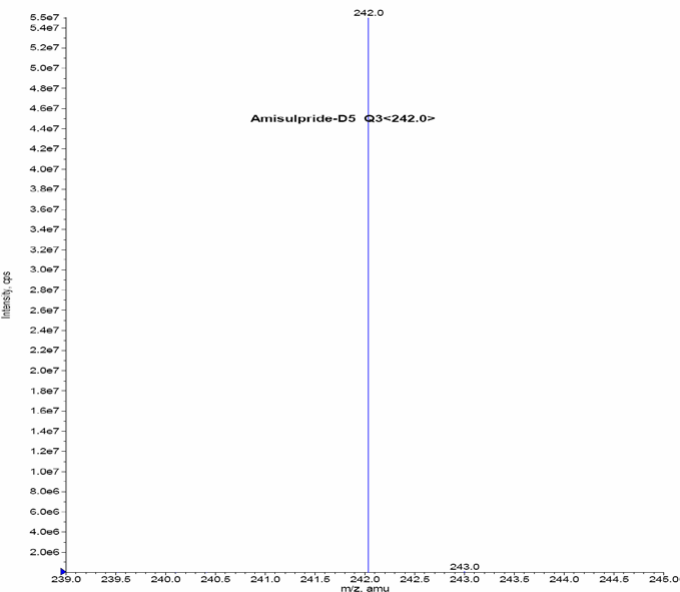
Mass spectrum of Amsulpride-*d*_5_ production

**Fig. 3a.**
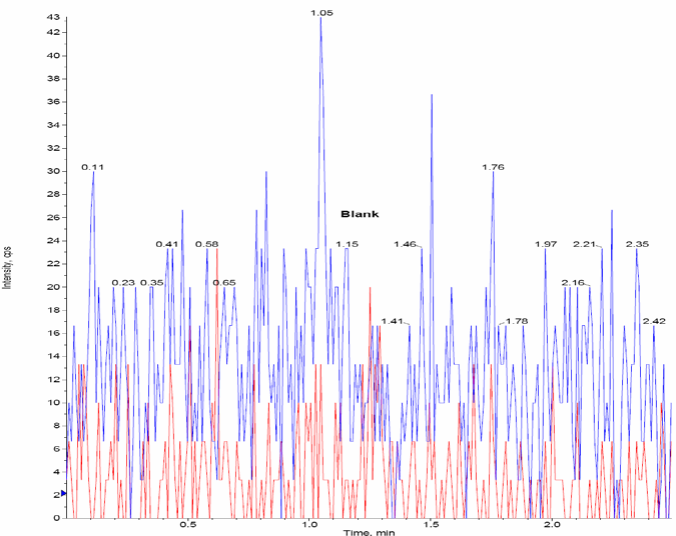
MRM Chromatogram of Blank Human Plasma

**Fig. 3b.**
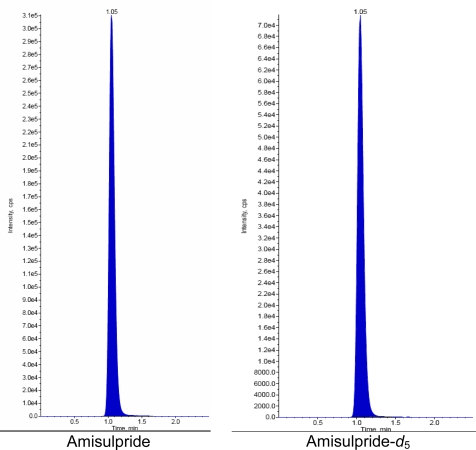
Chromatogram of LOQ

**Tab. 1.** Calibration curves from one batch of the validation section

**Spiked plasma concentration (ng/mL)**	**Concentration measured(mean) (ng/mL)±SD**	**CV (%) (*n* = 5)**	**Accuracy%**
2.00	2.03 ± 0.03	1.5	101.5
4.00	3.86 ± 0.15	3.9	96.5
10.00	10.09± 0.13	1.3	99.1
25.00	25.07 ± 0.68	2.7	99.7
250.00	252.26 ± 5.04	2.0	99.1
500.00	499.91 ± 6.48	1.3	100.0
1000.00	989.99 ± 18.14	1.8	99.0
1500.00	1514.93 ± 46.87	3.1	101.0
2000.00	2026.48 ± 43.40	2.1	101.3
2500.00	2464.29 ± 90.38	3.7	98.6

**Tab. 2.** Precision and accuracy (analysis with spiking plasma samples at four different concentrations)

**Spiked plasma concentration (ng/mL)**	**Within-run**			**Between-run**		

	**Concentration measured (*n*=6) (ng/mL) (mean ± S.D)**	**R.S.D.^a^ (%)**	**Accuracy %**	**Concentration measured (*n*=30) (ng/mL) (mean±S.D.)**	**R.S.D.^a^ (%)**	**Accuracy %**
2.00	1.99±0.20	1.6	99.50	1.97±0.18	1.5	98.42
6.00	5.90±0.10	1.7	98.3	5.76± 0.15	2.6	96.0
750.00	761.14±6.58	0.9	101.5	757.34± 11.57	1.5	101.0
1750.00	1746.15±20.44	1.2	99.8	1763.73±49.24	2.8	100.8

a $\frac{\text{Standard}\;\text{deviation}}{\text{Mean}\;\text{concentration}\;\text{measured}}\times 100$

**Tab. 3.** Stability of the samples

**Spiked plasma concentration (ng/mL)**	**Room temperature/Benchtop stability**		**Autosampler stability**	

	**25.0 h**		**99.0 h**	

	**Concentration measured (*n*=6) (ng/mL) (mean±S.D)**	**(*n*=6) CV(%)**	**Concentration measured (*n*=6) (ng/mL) (mean±S.D)**	**(*n*=6) CV(%)**

6.00	5.68 ± 0.13	2.4	6.29 ± 0.14	2.2
1750.00	1801.90 ± 22.31	1.2	1818.89 ± 24.78	1.4

**Spiked plasma concentration (ng/mL)**	**Long term stability**		**Freeze and thaw stability**	

	**55 days**		**Cycle 3 (48 h)**	

	**Concentration measured (*n*=6) (ng/mL) (mean±S.D)**	**(*n*=6) CV(%)**	**Concentration measured (*n*=6) (ng/mL) (mean±S.D)**	**(*n*=6) CV(%)**

6.00	5.59 ± 0.04	0.7	5.94 ± 0.4	7.3
1750.00	1807.13 ± 21.13	1.2	1820.14 ± 25.99	1.4

**Tab. 4.** Mean pharmacokinetic parameters of Amisulpride in 10 healthy volunteers after oral administration of 50 mg test and reference products.

**Amisulpride**	**Pharmacokinetic Parameter**			
	**Cmax (ng/ mL)**	**Tmax (hr)**	**AUC_0-t_ (ng. hr/mL)**	**AUC_0-∞_ (ng. hr/mL)**
Test	947.9	3	10710.8	10864.3
Reference	1093.4	3	11660.4	11817.7

AUC_0-∞_…area under the curve extrapolated to infinity;

AUC_0-t_…area under the curve up to the last sampling time;

Cmax…the maximum plasma concentration;

Tmax…the time to reach peak concentration.

**Tab. 5.** Test/Reference values for log-transformed pharmacokinetic parameters of Amisulpride after oral administration of 50 mg of test and reference products in 10 healthy male volunteers

	Pharmacokinetic Parameter		
	Cmax (ng /mL)	AUC_0-t_ (ng. hr/mL)	AUC_0-∞_ (ng. hr/mL)
Test/Reference	86.7	91.8	91.9
